# DATASET for validation the relationship between workplace spirituality, organizational commitment, and workplace deviance

**DOI:** 10.1016/j.dib.2020.105872

**Published:** 2020-06-17

**Authors:** Shofia Amin, Zulfina Adriani, Khaeruddin, Akhmad Habibi

**Affiliations:** a Faculty Economics and Bussiness, Universitas Jambi, Indonesia; b Lembaga Pengelola Dana Pendidikan (LPDP), Indonesia; c Faculty of Education and Teacher Training, Universitas Jambi, Indonesia; d Faculty of Education and Arts, School of Education, The University of Newcastle, Australia

**Keywords:** Civil servant, Workplace deviance, Workplace spirituality, Organizational commitment

## Abstract

The current dataset examines the relationship between workplace spirituality and workplace deviance through the improvement of organizational commitment. The instruments from previous studies were adapted and validated through content validity. Further, it was translated from English to Indonesian language. In the data preparation, the computation of Skewness and Kurtosis, as well as Histogram, was done. Reliability assessment was done through Cronbach's alpha. Exploratory Factor Analysis (EFA) and Confirmatory Factor Analysis (CFA) were addressed for the three constructs; workplace spirituality, organizational commitment, and workplace deviance. In an academic standpoint, the dataset can extend in-depth contributions and references for further researchers as a basis of the empirical evidence in relation to the relationship between the workplace spirituality, organizational commitment, and the workplace deviance. It is also beneficial for a model for reducing the workplace deviance from employee perspectives in the context of developing countries. Access to this dataset may contribute to stakeholders in establishing policies to reduce the workplace deviance.

**Specifications Table**

**Table:** 

**Subject**	Management
**Specific subject area**	Human resource management; organizational behavior
**Type of data**	Table Figure
**How data were acquired**	Face and content validity, survey, and SEM AMOS
**Data format**	Raw Analyzed Filtered
**Parameters for data collection**	The instrument includes workplace spirituality, improvement of organizational commitment, and workplace deviance.
**Description of data collection**	The instruments from previous studies were adapted and validated through content validity. Further, it was translated from English to Indonesian language. In the data preparation, the computation of Skewness and Kurtosis, as well as Histogram, was done. Reliability assessment was done through Cronbach's alpha. Exploratory Factor Analysis (EFA) and Confirmatory Factor Analysis (CFA) were addressed for the three main constructs; workplace spirituality, improvement of organizational commitment, and workplace deviance.
**Data source location**	Region: Jambi Country: Indonesia Latitude and longitude (and GPS coordinates) for collected samples/data 1.6101° S, 103.6131° E
**Data accessibility**	On a public repository: Repository name: Mendeley Data Data identification number: DOI: 10.17632/79y9ntcxzs.1 Direct URL to data: https://data.mendeley.com/datasets/79y9ntcxzs/1

**Value of the Data**

the dataset can extend in-depth contributions and references for further researchers as a basis of the empirical evidence in relation to the relationship between the workplace spirituality, organizational commitment, and the workplace deviance.
The dataset is beneficial for a model for reducing the workplace deviance from employee perspectives in the context of developing countries
Access to this dataset may contribute to stakeholders in establishing policies to reduce the workplace deviance

## 1 Data description

**Fig. 1:**
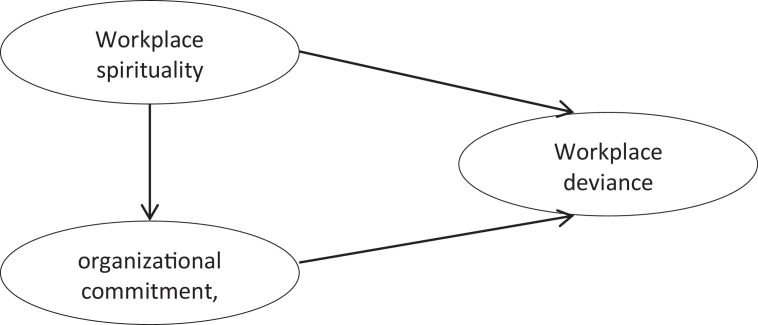
Proposed model.

Data were adapted from previous related studies [1], [2], [3]. Data of this survey study include three primary constructs, namely workplace spirituality, organizational commitment, and workplace deviance. Workplace spirituality include three sub-constructs; meaningful work (6 items), sense of community (8 items), alignment with organization's value (7 items). In addition, organizational commitment refers to three sub-constructs e.g. affective (8 items), normative (7 items), and continuance (8 items). Finally, workplace deviance contains two sub-constructs; interpersonal (7 items) and organizational (11 items). A 5-scale Likert scale (1 = Strongly Disagree; 5 = Strongly Agree) was used for workplace spirituality and organizational commitment. Meanwhile, we reversed the scale for working deviance (1 = Strongly Agree; 5 = Strongly Disagree). The proposed model of the relationship among constructs in this study is informed in Fig 1. A summary of data presented in this dataset is shown in Table 1, Table 2, Table 3, Table 4. Table 1 informs EFA result of workplace spirituality; Table 2 performs EFA result of organizational commitment; and Table 3 describes EFA result of workplace deviance. In addition, the CFA results of the three constructs are shown in Table 4.

**Table 1:** EFA Result; workplace spirituality.

Sub construct	Item	eigenvalue	communality	Cross loading		
Alignment with organization's value	AOV1	4.371	.661	.784		
	AOV7		.590	.764		
	AOV3		.556	.743		
	AOV2		.563	.733		
	AOV6		.505	.689		
	AOV5		.468	.652		
Meaningful work	MW3	2.942	.698		.833	
	MW4		.626		.784	
	MW2		.634		.772	
	MW1		.554		.738	
Sense of community	SC3	1.699	.615			.715
	SC4		.542			.678
	SC6		.554			.672
	SC2		.525			.653
	SC1		.380			.607
	SC7		.539			.410

**Table 2:** EFA Result; organizational commitment.

Sub construct	Item	Eigenvalue	Communality	Cross loading		
Normative	N3	5.794	.715	.843		
	N4		.694	.774		
	N1		.603	.750		
	N2		.567	.731		
	N7		.671	.731		
	N5		.543	.705		
	N6		.539	.641		
Affective	A7	3.051	.494		.696	
	A1		.542		.685	
	A2		.436		.630	
	A5		.332		.552	
	A4		.545		.510	
	A8		.317		.508	
	A3		.294		.481	
	A6		.387		.464	
Continuance	C2	2.184	.588			.737
	C3		.647			.730
	C1		.365			.589
	C8		.465			.589
	C4		.460			.557
	C5		.525			.531
	C7		.300			.408

**Table 3:** EFA Result; workplace deviance.

Sub construct	Item	Eigenvalue	Communality	Cross loading	
Organizational	O5	3.902	.634	.796	
	O9		.497	.667	
	O3		.438	.654	
	O6		.503	.643	
	O8		.452	.625	
	O4		.308	.552	
	O1		335	.512	
Interpersonal	I7	2.034	.730		.854
	I5		.722		.830
	I1		.505		.710
	I3		.588		.656
	I4		.324		.470

**Table 4:** CFA assessment values.

Construct	Loading range	(χ2)	CFI	TLI	RMSEA	Sub construct	CR	AVE	α
Workplace spirituality	.570–0.830	*p* > 0.050	.931	.911	.076	Alignment with organization's value	.731	.693	.858
						Meaningful work	.796	.746	.762
						Sense of community	.731	.635	.735
Organizational commitment	520–0.830	*p* > 0.050	.925	.907	.078	Normative	.784	.725	.862
						Affective	.725	.683	.838
						Continuance	.661	.623	.703
Workplace deviance	550–0.890	*p* > 0.050	.969	.945	.079	Organizational	.772	.723	.758
						Interpersonal	.803	.707	.807

## 2 Experimental design, materials, and methods

The items were validated through content validity [4,5]. Five experts in Human resource management and organizational behaviour were invited to discuss all items for context and setting evaluation. On this stage, two items on workplace deviance were dropped; it was recommended by more than 50% of the experts. Back translation proposed by [6] was done before the distribution of the questionnaire.

The questionnaire was distributed to 350 Indonesian government employees in Jambi. Three hundred and fifteen responses were analysed; Thirty employees did not return the questionnaire while five responses were not completed. For the data preparation, Skewness and Kurtosis values of each construct were found to be normal, ranging from −1 to +1 for the Skewness and −2 to +2 for the Kurtosis [7]. Using histogram, the data were reported to be normally distributed. Cronbach's alpha for all constructs extends 0.700 (acceptable).

For the EFA, component principal analysis (PCA) approach was used to formulate uncorrelated linear combination against observable constructs; Kaiser Meyer Olkin (>0.500), Bartlett's Test of Sphericity (*p* < 0.05), eigenvalue (factor = > 1.0), communality (>0.30), and factor loading (>.0 40) [7]. For workplace spirituality with Varimax rotation, three factors were achieved. Kaiser Meyer Olkin (0.743) and Bartlett's Test of Sphericity (*p* = 0.000) exceed the threshold values. Table 1 informs the eigenvalue, communality, and cross-loading of the sub-constructs. Some items were deleted due to low loading and cross-loading as well as low communality values. The deleted items were MW5, MW6, AOV4, SC5, and SC8. For organizational commitment, three factors are informed; Normative, Affective, and Continuance. Kaiser Meyer Olkin (0.756) and Bartlett's Test of Sphericity (*p* = 0.000) values are also acceptable. The eigenvalue, communality, and cross-loading of the sub-constructs of organizational commitment are shown in Table 2. One item (C6) was deleted from organizational commitment. Finally, workplace deviance's refers to two factors which the Kaiser Meyer Olkin is satisfactory (0.732). Similarly, its Bartlett's Test of Sphericity extends the required score (*p* = 0.000). A complete elaboration of the eigenvalue, communality, and cross-loading is reported in Table 3. Several items; O2, O7, O10, O11, I2, I6, were dropped due to low loading values and cross-loading [8].

**Fig. 2:**
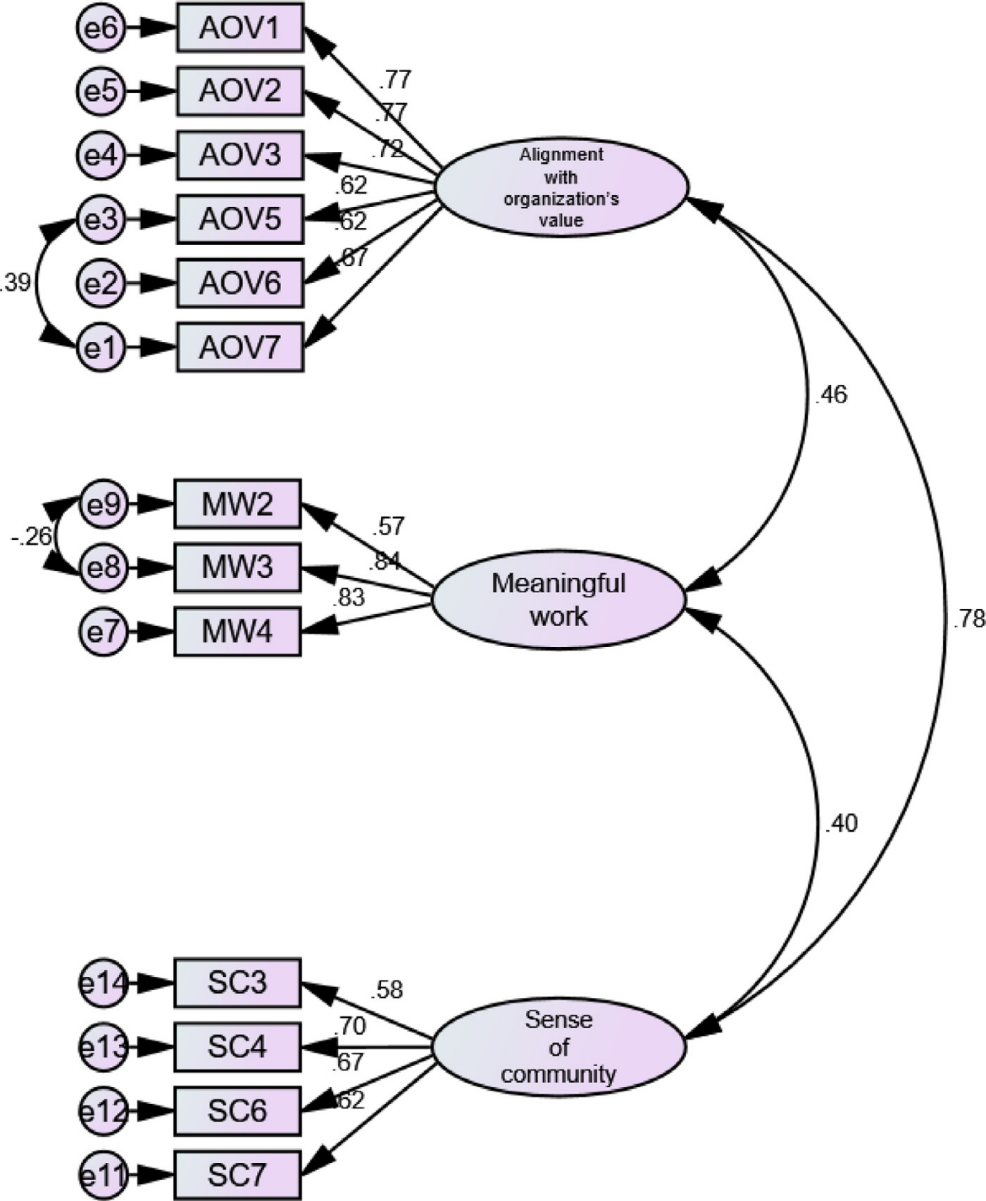
CFA of workplace spirituality.

**Fig. 3:**
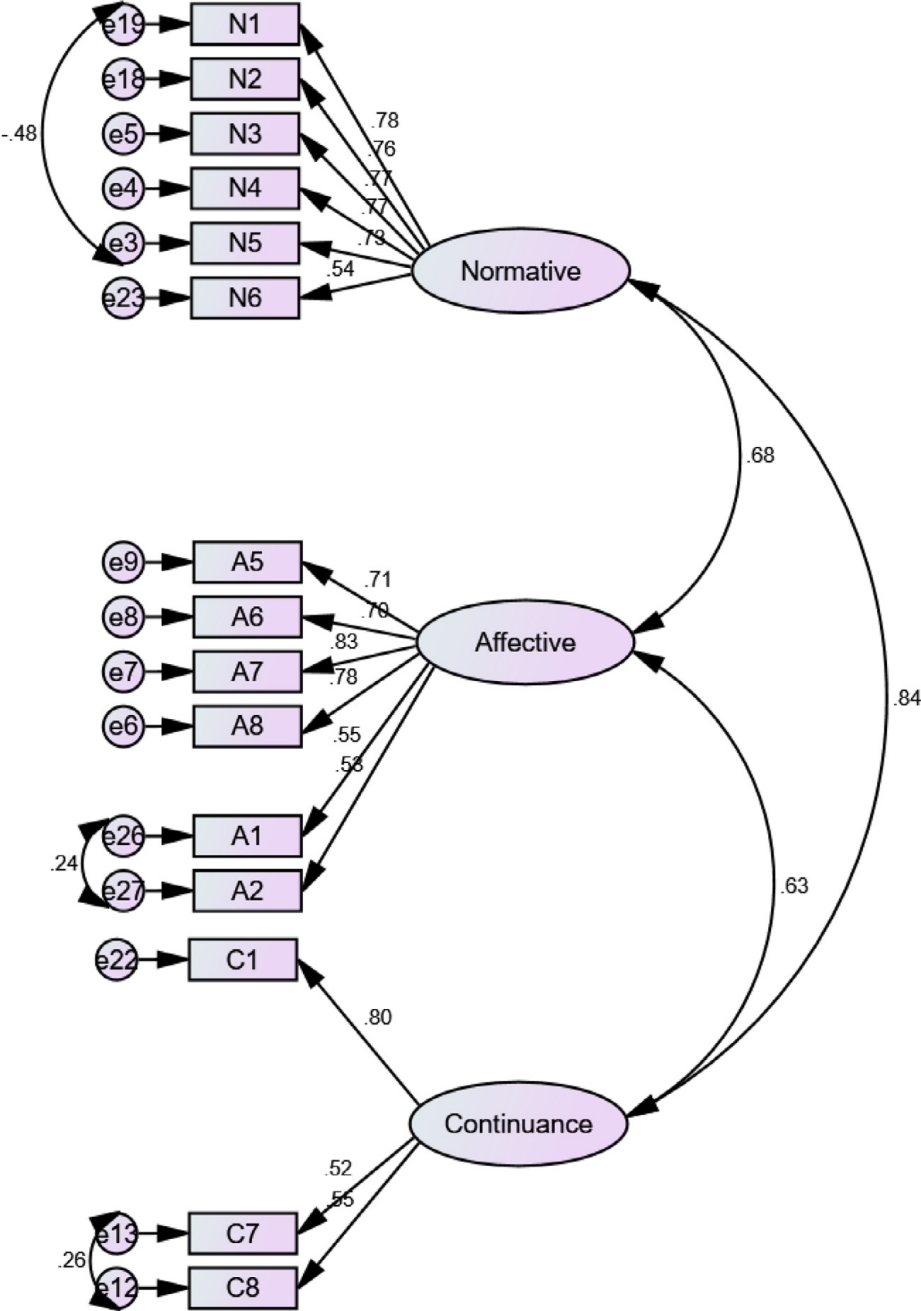
CFA of organizational commitment.

**Fig. 4:**
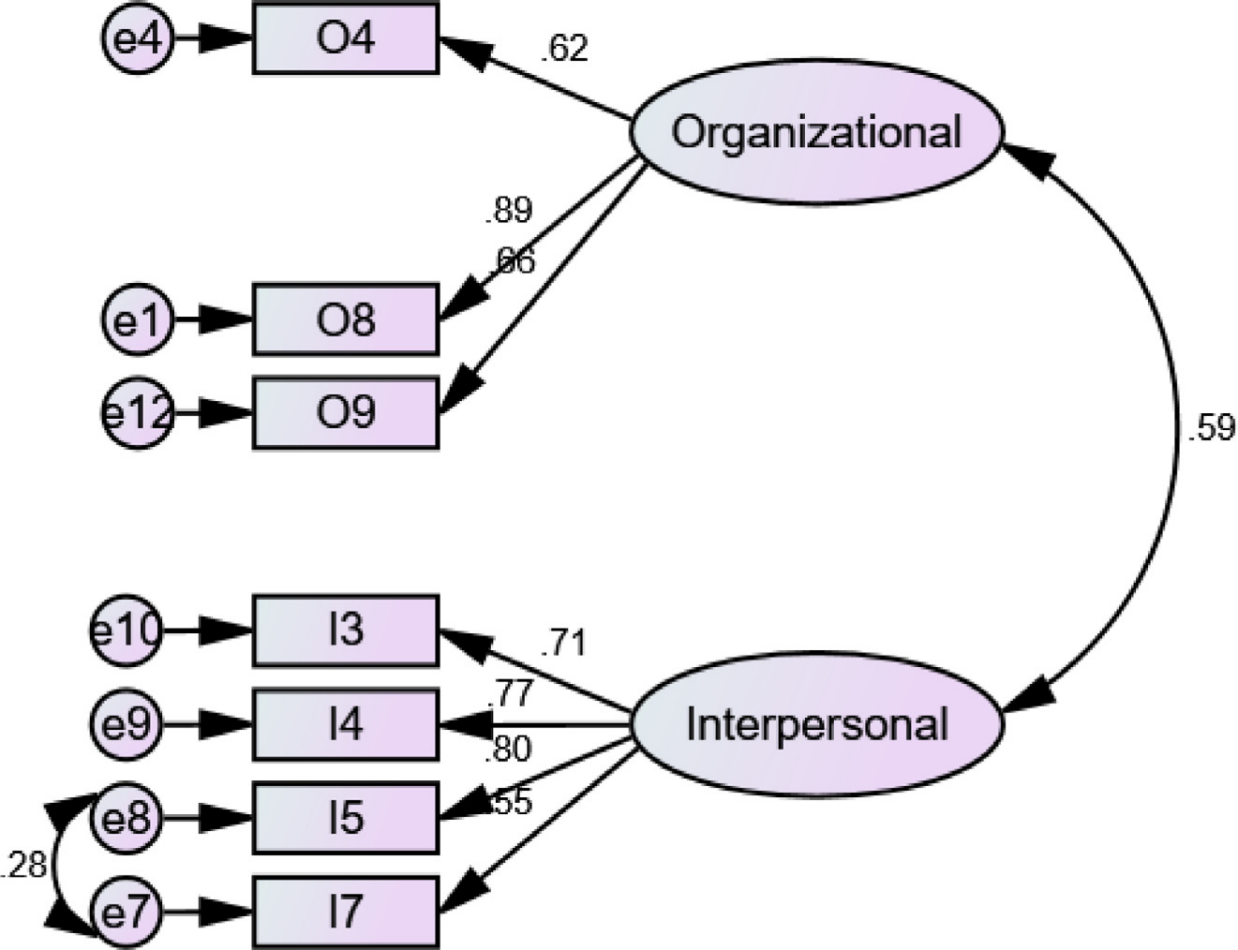
CFA of working deviance.

Confirmatory Factor Analysis (CFA) steps was computed in AMOS 23.0. Goodness of fit is assessed using the chi-square (χ2) (*p* > 0.050), the comparative fit index (CFI >0.90), the Tucker–Lewis index (TLI> 0.90), as well as the root mean-square error of approximation (RMSEA < 0.08) [7,9]. The Cronbach's alpha coefficients, Composite Reliability (CR), and Average Variance Extracted (AVE) were implemented in calculating the reliability of the questionnaire. Alpha should be ranging of 0.60–0.70 in exploratory research [7]. CR should not be less than 0.60, and AVE should not be less than 0.50 [10]. For CFA,  standardized loading estimates should be 0.50 or more. The initial measurements of the three CFA processes did not achieve the fit model. Some items were dropped since they have low loading and some modifications by drawing covariance among error variances were applied (Figs. 1–3). All values of constructs and sub-constructs through the CFA process have met the cut off values (Table 4). All loadings value are above 0.50 as the standardized cut off value (Figs. 2–4)

## Declaration of Competing Interest

The authors declare that they have no known competing financial interests or personal relationships which have, or could be perceived to have, influenced the work reported in this article.

## Appendix Supplementary materials

Image, application 1
